# SIADH as an Underrecognized Manifestation of Porphyria-like Crises in Hereditary Tyrosinemia Type 1: Clinical and Pathophysiological Insights

**DOI:** 10.3390/ijms27020660

**Published:** 2026-01-09

**Authors:** Eleonora Saraceno, Ilaria Serra, Beatrice Bracci, Veronica Pagliardini, Michele Pinon, Gerdi Tuli, Antonia Versace, Claudia Bondone, Marco Spada

**Affiliations:** 1 Department of Public Health and Pediatrics, Postgraduate School of Pediatrics, Regina Margherita Children’s Hospital, University of Torino, 10126 Torino, Italy; 2 Department of Pediatrics, Regina Margherita Children’s Hospital, University of Torino, 10126 Torino, Italy; 3 Pediatric Emergency Department, Regina Margherita Children’s Hospital, 10126 Torino, Italy; 4 Pediatric Gastroenterology Unit, Regina Margherita Children’s Hospital, 10126 Torino, Italy; 5 Pediatric Endocrinology Unit, Regina Margherita Children’s Hospital, 10126 Torino, Italy

**Keywords:** hereditary tyrosinemia type 1, nitisinone, acute porphyria, SIADH, hyponatremia

## Abstract

Hereditary tyrosinemia type 1 (HT1) is a rare metabolic disorder caused by fumarylacetoacetate hydrolase deficiency, leading to the accumulation of toxic metabolites such as fumarylacetoacetate (FAA) and succinylacetone (SA). We report an 11-year-old boy with poorly controlled HT1 who presented with a severe neurovisceral crisis after suboptimal adherence to nitisinone (NTBC) therapy, characterized by abdominal pain, hypertension, paralytic ileus, seizures, and profound hyponatremia. Biochemical evaluation revealed markedly elevated urinary δ-aminolevulinic acid (ALA), consistent with a porphyria-like metabolic decompensation, together with inappropriately increased plasma copeptin in the setting of hypotonic hyponatremia and clinical euvolemia, fulfilling diagnostic criteria for the syndrome of inappropriate antidiuretic hormone secretion (SIADH). Optimization of NTBC therapy combined with tailored fluid management resulted in complete clinical and biochemical recovery. This case supports a pathophysiological link between acute disruption of the heme–porphyrin pathway and inappropriate antidiuretic hormone secretion. In HT1, this susceptibility may be further amplified by FAA- and SA-mediated oxidative stress, mitochondrial dysfunction, and heme depletion, with an additional contribution from SA-associated renal tubular impairment. Overall, our findings underscore SIADH as a potentially underrecognized cause of acute hyponatremia in HT1 and highlight the importance of strict NTBC adherence and early monitoring of urinary ALA during metabolic decompensation.

## 1. Introduction

Hereditary tyrosinemia type 1 (HT1) is a rare autosomal recessive disorder caused by pathogenic variants in the *FAH* gene, leading to deficiency of fumarylacetoacetate hydrolase (FAH), the final enzyme of tyrosine catabolism. The enzymatic block results in accumulation of the toxic metabolites—maleylacetoacetate, fumarylacetoacetate (FAA), and succinylacetone (SA)—that induce progressive hepatocellular injury, renal tubular dysfunction, and a markedly increased risk of hepatocellular carcinoma [1,2].

Nitisinone (NTBC), a potent inhibitor of 4-hydroxyphenylpyruvate dioxygenase, has dramatically improved survival by preventing upstream production of FAA and SA [3]. When combined with a low-protein diet supplemented with a phenylalanine- and tyrosine-free amino acid mixtures, NTBC ensures long-term preservation of hepatic and renal function in patients with good compliance. However, maintaining strict adherence—particularly during adolescence—represents a major clinical challenge [3,4].

Poor compliance is the leading cause of acute neurologic porphyria-like decompensation in HT1 [1,4,5,6], a life-threatening condition that shares key biochemical features with acute hepatic porphyrias. In particular, elevated succinylacetone (SA) levels potently inhibit δ-aminolevulinic acid dehydratase (ALAD), leading to systemic accumulation of δ-aminolevulinic acid (ALA) and functional disruption of the heme–porphyrin pathway [2,7]. As in acute porphyrias, this metabolic derangement results in neurovisceral and autonomic manifestations [8], including anorexia, poorly localized abdominal or limb pain, axial or extensor hypertonia, weakness that may progress to respiratory failure, autonomic instability, hypertension, and hyponatremia with possible seizures [1]. Among these features, hyponatremia remains one of the least understood.

The syndrome of inappropriate antidiuretic hormone secretion (SIADH) is defined by non-osmotic arginine vasopressin (AVP) release leading to impaired renal free-water excretion and dilutional hyponatremia [9]. SIADH has been reported as a cause of hyponatremia during acute porphyric attacks, where it has been attributed to intense visceral pain, hypothalamic stimulation, or paralytic ileus [10].

Here, we describe an 11-year-old patient with poorly controlled HT1 who presented with profound hyponatremia and seizure due to SIADH during an acute porphyria-like episode. We further propose a mechanistic framework whereby FAA- and SA-mediated toxicity may promote ALA accumulation, oxidative stress, mitochondrial dysfunction, and systemic heme depletion [2], thereby potentially predisposing to dysregulation of AVP secretion during acute disruption of the heme–porphyrin pathway and, more specifically, during HT1 decompensation. This integrated clinical and pathophysiological perspective may refine current understanding of acute HT1 crises and carries relevant implications for their clinical management.

## 2. Case Presentation

An 11-year-old boy (weight 32 kg) with genetically confirmed HT1 was admitted to our Pediatric Emergency Department after experiencing a generalized tonic seizure. His past medical history was remarkable for classical early-onset HT1, diagnosed at 3 months of age, when he presented with subacute hepatitis, markedly impaired hepatic synthetic function, and respiratory insufficiency attributed to neuropathy related to a porphyria-like crisis. Despite a delayed clinical response to NTBC therapy, his liver function gradually improved, α-fetoprotein levels normalized, and no focal hepatic lesions were detected at his most recent abdominal ultrasound. At the time of admission, he was receiving NTBC at a total daily dose of 30 mg (≈1 mg/kg/die) along with a protein-restricted diet (Table 1).

During the week preceding admission, the patient developed acute abdominal pain, constipation, anorexia, and two episodes of vomiting. He was evaluated twice in the Emergency Department; laboratory tests were unremarkable, and he was discharged on both occasions.

Two days after his last evaluation, he experienced a sudden loss of consciousness with tonic posturing. Upon arrival, vital signs were stable except for marked hypertension (140/90 mmHg), and neurological examination was unremarkable, with no focal motor deficits and no clinical signs of acute flaccid weakness or peripheral neuropathy. The patient was clinically euvolemic, with no signs of dehydration or fluid overload. Laboratory investigations revealed profound electrolyte derangement, with severe hyponatremia (106 mmol/L), hypokalemia (3.1 mmol/L), and hypochloremia (65 mmol/L). Liver and renal function tests, as well as inflammatory markers, were within normal ranges.

During Emergency Department observation, he developed a second convulsive episode, which promptly resolved after intravenous midazolam administration. He was subsequently transferred to the Pediatric Intensive Care Unit, where electrolyte abnormalities were corrected under close monitoring with carefully titrated isotonic saline infusions, following a controlled sodium correction target not exceeding 6–8 mmol/L per 24 h. Electroencephalography (EEG) showed diffuse background slowing consistent with metabolic encephalopathy. Computed tomography of the brain and abdomen revealed no acute structural abnormalities but demonstrated paralytic ileus.

Endocrine evaluation showed normal adrenal and thyroid function. Plasma copeptin was elevated at 20 pmol/L (reference range 3–8 pmol/L) despite hypotonic hyponatremia, and urinary studies revealed inappropriately high urinary sodium excretion. Together, these findings supported the diagnosis of SIADH [6] (Table 1).

Accordingly, the fluid management strategy was promptly revised: free-water intake was restricted, and maintenance fluids were reduced accordingly.

**Figure 1:**
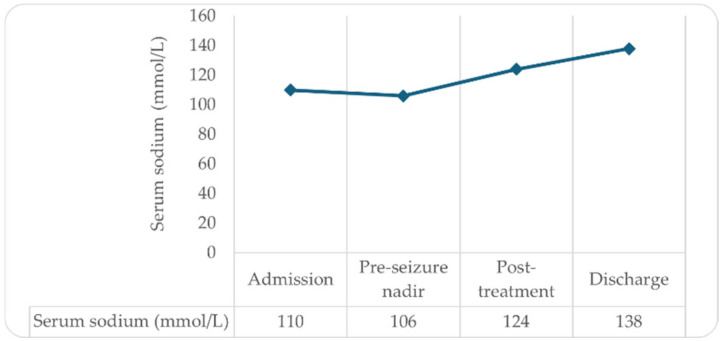
Time course of serum sodium during acute metabolic decompensation. Severe hypotonic hyponatremia coincided with neurogenic abdominal pain and two seizures during a period of poor Nitisinone (NTBC) adherence. Serum sodium progressively corrected following fluid restriction, NTBC reintroduction, and stabilization of metabolic parameters.

Over the subsequent days, frequent biochemical and neurological assessments ensured gradual normalization of serum sodium without overcorrection or osmotic complications (Figure 1).

However, the patient continued to experience abdominal pain, for which no surgical intervention was required, and blood pressure remained elevated despite tapering of intravenous hydration. Additional history obtained after stabilization revealed recent weight gain without NTBC dose adjustment. Importantly, the patient’s mother later discovered that he had been hiding his NTBC tablets behind a piece of furniture, indicating a prolonged, previously unrecognized period of poor adherence.

Given the clinical constellation—neurovisceral pain, hypertension, ileus, seizures, and SIADH—an acute porphyria-like crisis was strongly suspected. A careful review of the patient’s medication history did not reveal exposure to known porphyrinogenic drugs prior to the acute event. In the context of fluctuating adherence to NTBC, this episode was interpreted as a metabolic decompensation. Consistently, urinary ALA was found markedly elevated (17.47 mg/g creatinine), confirming the clinical suspicion. On this basis, NTBC dosage was therefore increased to 50 mg/die (≈1.5 mg/kg/die)

The patient’s symptoms subsequently improved. By the time of discharge (day 14), serum electrolytes had normalized, urinary ALA had decreased to 5.2 mg/g creatinine, the EEG had returned to baseline, and overall clinical status was stable.

## 3. Discussion

Acute neurologic porphyria-like crises represent a well-recognized but still incompletely defined complication of HT1. Their clinical manifestations—neurogenic abdominal pain, autonomic instability, ileus, hypertension, and seizures—are well documented, particularly in patients with suboptimal NTBC exposure [1]. Hyponatremia is also frequently reported during acute metabolic decompensation [1,11], yet its underlying mechanisms in HT1 remain poorly defined.

In acute hepatic porphyrias, hyponatremia is often attributed to SIADH, triggered by severe visceral pain or intestinal fluid sequestration during paralytic ileus [10]. While similar mechanisms may also contribute to HT1, more direct metabolic pathways may underlie AVP dysregulation during acute HT1 decompensation.

To date, only one previous report has described SIADH during a porphyria-like crisis in HT1 [12], raising the possibility that inappropriate AVP secretion may constitute an underrecognized component of the acute metabolic phenotype.

The toxic metabolites that accumulate in FAH deficiency are central drivers of cellular dysfunction in HT1 [2].

Specifically, FAA is a highly reactive compound that induces oxidative stress, depletes intracellular glutathione, and triggers ERK-dependent pathways, promoting mitochondrial dysfunction, DNA damage, and hepatocarcinogenesis [2].

SA, a downstream derivative of FAA and biochemical hallmark of HT1, adds a further layer of metabolic disruption by potently inhibiting ALAD, the second enzyme of heme biosynthesis [2]. ALAD inhibition reproduces the biochemical defect of ALAD-deficiency porphyria, leading to systemic and urinary accumulation of ALA [7,8].

As a consequence of heme–porphyrin pathway disruption, up to 40% of children with HT1 were reported to experience porphyria-like neurovisceral crises in the pre-NTBC era [5]. Although these events have become less frequent with NTBC therapy [3], current clinical guidelines continue to acknowledge their occurrence during periods of insufficient NTBC exposure, including undertreatment, poor compliance, or therapy interruptions of variable duration [1,6,8,11]. However, robust contemporary epidemiological data on the incidence of acute porphyria-like crises in HT1 are lacking, as such events are rarely reported in well-controlled cohorts, suggesting that they may remain under-recognized despite NTBC markedly reducing—but not abolishing—susceptibility to metabolic derangement.

ALA accumulation exerts direct neurotoxic effects: it generates reactive oxygen species, disrupts iron homeostasis by mobilizing labile iron from ferritin, induces mitochondrial membrane depolarization, and impairs ATP production. Concurrently, ALA-mediated heme depletion reduces the activity of heme-dependent enzymes critical for redox homeostasis, neurotransmitter biosynthesis, and osmoregulatory signaling [2,13,14].

Although acute flaccid weakness is a recognized feature of acute hepatic porphyrias, occurring in approximately 10–60% of patients during an acute attack, the relationship between ALAD inhibition, ALA accumulation, and neurological phenotype is complex. Rapidly evolving neuropathy may mimic Guillain–Barré syndrome; however, preceding visceral pain or other neurovisceral manifestations often signal an emerging porphyric neuropathy. Neurovisceral and autonomic symptoms may therefore precede—or occur independently of—overt motor deficits, likely reflecting preferential exposure of autonomic and enteric nervous system structures due to regional differences in blood–nerve and blood–brain barrier permeability [15]. In this context, abdominal pain—classically the most frequent presenting symptom of acute hepatic porphyrias [1]—may represent an early clinical warning sign of metabolic decompensation in HT1. This susceptibility may be further amplified by chronic metabolic stress from sustained SA and FAA exposure, which, together with individual variability in neuronal and autonomic sensitivity, may modulate ALA neurotoxicity and contribute to phenotypic heterogeneity. Within this pathophysiological framework, dysregulation of AVP secretion may represent a plausible downstream effect of ALA-mediated injury rather than an epiphenomenon.

**Figure 2:**
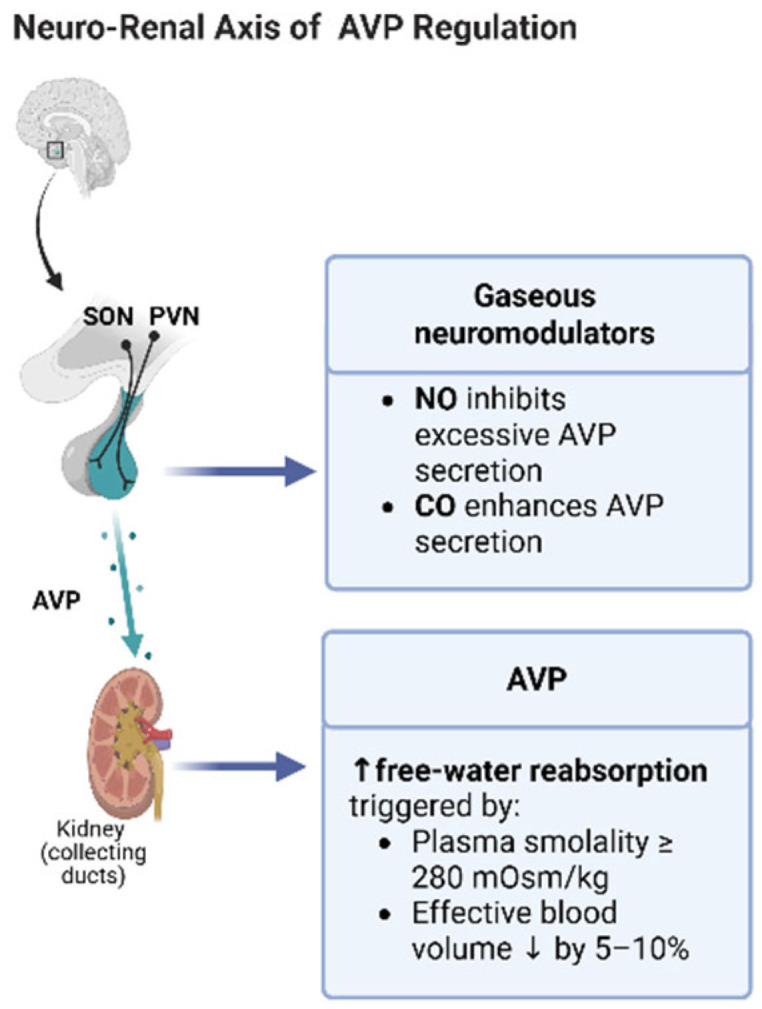
Neuro–renal integration of arginine vasopressin (AVP) secretion and renal water handling. Schematic representation of the hypothalamic supraoptic and paraventricular nuclei (SON/PVN), where gaseous neuromodulators regulate magnocellular neuron (MNC) excitability: nitric oxide (NO) exerts an inhibitory tone that prevents excessive AVP release, whereas carbon monoxide (CO) enhances AVP secretion. AVP released from the posterior pituitary acts on renal collecting ducts to increase free-water reabsorption when plasma osmolality exceeds ~280 mOsm/kg or when effective arterial blood volume falls by 5–10%. Abbreviations: AVP, arginine vasopressin; CO, carbon monoxide; NO, nitric oxide; PVN, paraventricular nucleus; SON, supraoptic nucleus. Created by Biorender.com.

Magnocellular neurons (MNCs) of the supraoptic (SON) and paraventricular (PVN) nuclei—responsible for AVP and oxytocin release—are highly metabolically active cells, requiring intact mitochondrial ATP production as well as heme-dependent enzymatic activity to maintain normal electrophysiological function [16,17]. The gaseous neuromodulators nitric oxide (NO) and carbon monoxide (CO), produced, respectively, by heme-dependent nitric oxide synthases (NOSs) and heme oxygenase (HO), exert essential and opposing regulatory effects on MNC excitability. NO provides a tonic inhibitory influence that prevents excessive or inappropriate AVP release [18]. Conversely, CO enhances MNC excitability and promotes AVP secretion, particularly under osmotic stress [19]. The neuro–renal axis of AVP secretion and renal water handling is summarized schematically in Figure 2.

NOS activity is strictly heme-dependent. In animal and cellular models, reduced heme availability or oxidative stress can uncouple NOS, decreasing inhibitory NO signaling while increasing superoxide production [20,21,22].

In parallel, oxidative stress upregulates HO, increasing CO production [19]. However, under SA-mediated inhibition of heme synthesis, HO-1 induction may further deplete residual heme pools [23], thereby reinforcing NOS uncoupling and establishing a self-amplifying redox loop characterized by persistent oxidative stress and impaired gaseous neuromodulation. Collectively, these processes may shift MNCs toward a hyperexcitable state even in the absence of osmotic triggers.

Additional mechanisms may further amplify this vulnerability. ALA interferes with presynaptic GABA_A_ autoreceptor function, disrupting GABAergic inhibition within key hypothalamic neuroendocrine nuclei and thereby promoting inappropriate AVP release. Moreover, ALA-induced mitochondrial dysfunction in MNCs may interfere with osmosensory signal transduction, rendering these neurons abnormally sensitive to minor osmotic fluctuations [13].

Although these pathways have not been specifically studied in HT1, the shared biochemical landscape of HT1 and acute hepatic porphyrias [7,24] provides a plausible, biologically grounded explanation for AVP dysregulation during acute metabolic instability in HT1.

Importantly, HT1 may confer a deeper, disease-specific metabolic vulnerability: unlike acute porphyrias, where ALA accumulation is episodic, HT1 is characterized by chronic ALAD inhibition, persistent oxidative stress, and continuous mitochondrial injury due to FAA and SA exposure [2]. This chronic metabolic burden may predispose HT1 patients to more pronounced or prolonged disturbances in osmoregulatory pathways during acute decompensation.

Peripheral factors in HT1 further amplify this susceptibility: SA-mediated proximal tubular dysfunction may impair solute reabsorption, potentially compromising free-water handling by the kidney and thereby amplifying the antidiuretic effects of even modest AVP elevations [2,25,26].

**Figure 3:**
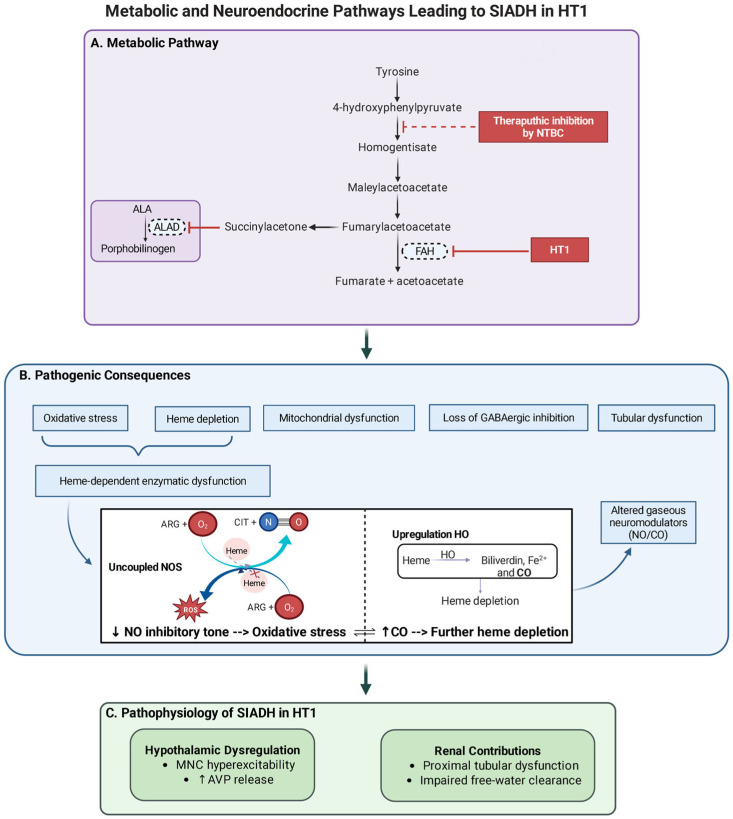
Integrated metabolic and neuroendocrine model linking hereditary tyrosinemia type 1 to inappropriate antidiuretic hormone secretion (SIADH). (**A**) FAH deficiency disrupts tyrosine catabolism, resulting in the accumulation of fumarylacetoacetate (FAA), succinylacetone (SA), and δ-aminolevulinic acid (ALA). (**B**) The accumulated metabolites impose converging metabolic stresses, including heme depletion, oxidative stress, impaired GABAergic inhibition, and mitochondrial dysfunction. In magnocellular neurons (MNCs), limited heme availability (indicated by the red “X” symbol in the NO pathway) and an oxidative intracellular environment promote nitric oxide synthase (NOS) uncoupling, resulting in reduced NO bioavailability and increased reactive oxygen species generation. Oxidative stress concurrently induces heme oxygenase (HO) activity, increasing CO production while further depleting intracellular heme pools. This progressive heme depletion exacerbates NOS uncoupling, establishing a self-amplifying redox loop characterized by sustained oxidative stress and impaired gaseous neuromodulation. The resulting disruption of the CO/NO balance compromises inhibitory control of AVP-secreting neurons within the supraoptic and paraventricular nuclei. In parallel, SA-mediated proximal tubular dysfunction reduces renal free-water clearance, further contributing to hyponatremia. (**C**) The central dysregulation of AVP release—characterized by MNC hyperexcitability and loss of inhibitory control—combined with impaired renal water handling creates a permissive environment for inappropriate AVP secretion, providing a mechanistic framework for SIADH development during porphyria-like crises in HT1. Abbreviations: ALA, δ-aminolevulinic acid; ALAD, δ-aminolevulinic acid dehydratase; AVP, arginine vasopressin; CO, carbon monoxide; FAH, fumarylacetoacetate hydrolase; HO, heme oxygenase; HT1, hereditary tyrosinemia type 1; MNC, magnocellular neuron; NO, nitric oxide; NOS, nitric oxide synthase; NTBC, nitisinone; SIADH, syndrome of inappropriate antidiuretic hormone secretion. Created with BioRender.com.

Figure 3 provides an integrated overview of the proposed pathophysiological model.

Taken together, these mechanisms provide a coherent and biologically plausible framework in which SIADH can be viewed as a clinical expression of the acute porphyria-like attacks—a feature shared across different conditions characterized by acute disruption of the heme–porphyrin pathway—rather than as a purely secondary or incidental complication, although dedicated mechanistic studies are still lacking.

Within this broader framework, SIADH in HT1 may represent not merely an extension of the ALA-driven model described in acute hepatic porphyrias, but rather the manifestation of a deeper, disease-specific vulnerability intrinsic to the metabolic architecture of HT1 itself.

Our patient developed SIADH following a prolonged period of poor NTBC adherence, presenting with severe neurogenic abdominal pain, paralytic ileus, hypertension, markedly elevated copeptin levels, increased urinary ALA excretion, and profound hypotonic hyponatremia, culminating in a seizure. The clinical and biochemical profile fulfilled established diagnostic criteria for SIADH [9], underscoring the importance of considering SIADH in the differential diagnosis of acute hyponatremia in HT1.

Importantly, recognizing SIADH in the context of an acute porphyria attack has direct therapeutic consequences. While glucose-containing fluids are standard treatment in acute porphyrias due to their ability to suppress hepatic ALA synthase [10], they may exacerbate hyponatremia in SIADH by increasing free-water retention [10,27]. In our patient, correct identification of SIADH guided careful modulation of isotonic saline therapy and prevented iatrogenic worsening of hyponatremia.

## 4. Conclusions

This case provides evidence that SIADH may represent a shared clinical expression of acute porphyria-like attacks, irrespective of the underlying metabolic trigger. In HT1, this susceptibility appears to be further amplified by disease-specific metabolic vulnerabilities.

In this setting, the clinical association of neurovisceral symptoms, profound hypotonic hyponatremia, and elevated copeptin levels supports the presence of inappropriate AVP secretion [9] in the setting of acute metabolic instability, potentially mediated by the neurotoxic effects of ALA, FAA, and SA.

Recognition of SIADH in this context carries immediate therapeutic implications, particularly regarding fluid management, which differs substantially from the glucose-based approach used in acute porphyria attacks [10].

Routine monitoring of urinary ALA emerges as a practical tool to assess NTBC adherence, detect early metabolic deterioration, and prevent life-threatening neurologic complications.

## Figures and Tables

**Table 1:** Clinical, biochemical, and management features of the patient during porphyria-like decompensation.

Category	Variable	Finding
Clinical Context	Age/Sex	11 yr/Male
	Nitisinone (NTBC) dose	1 mg/kg/die
	*FAH* genotype	c.709C>T/c.1025C>T
Clinical Presentation	Neurovisceral Pain	Abdominal pain, irritability, ileus-like episode
	Seizures	Generalized tonic–clonic seizures (two episodes)
	Blood Pressure	140/90 mmHg
Biochemical Evaluation	Creatinine (mg/dL)	0.5 (reference value 0.43–0.67)
	AST (U/L)	20 (reference value 10–40)
	ALT (U/L)	33 (reference value 10–45)
	Serum Sodium (mmol/L)	110 (reference value 135–145)
	Serum Osmolality (mOsm/kg)	219 (reference value 278–305)
	Urine Sodium (mmol/L)	73 (reference value < 30)
	Copeptin (pmol/L)	20 (reference value 3–8)
	Urinary δ-aminolevulinic acid (ALA) (mg/g crea)	17.47 (reference value < 4.5)
Management	Acute treatment	Controlled isotonic correction
	NTBC dose adjustment	1.5 mg/kg/die
Outcomes	Clinical Course	Rapid clinical stabilization
	Urinary ALA (mg/g crea)	5.2 (reference value 3–8)
	Blood pressure at discharge	Normalized

## Data Availability

The datasets generated for this study are not publicly available due to ethical and privacy restrictions related to individual clinical data, but are available from the corresponding author upon reasonable request.

## Abbreviations

The following abbreviations are used in this manuscript:

ALA	δ-aminolevulinic acid
ALAD	δ-aminolevulinic acid dehydratase
AVP	Arginine vasopressin
CO	Carbon monoxide
EEG	Electroencephalography
FAA	Fumarylacetoacetate
FAH	Fumarylacetoacetate hydrolase
HO	Heme oxygenase
HT1	Hereditary tyrosinemia type 1
MNC	Magnocellular neuron
NO	Nitric oxide
NOS	Nitric oxide synthase
NTBC	Nitisinone
PVN	Paraventricular nuclei
SA	Succinylacetone
SIADH	Syndrome of inappropriate antidiuretic hormone secretion
SON	Supraoptic nuclei
